# Bilateral Earlobe Creases and Subsequent Malignant Cerebral Infarction: A Patient With Diffuse Endothelial Dysfunction

**DOI:** 10.7759/cureus.9329

**Published:** 2020-07-21

**Authors:** Antonio Cruz Culebras

**Affiliations:** 1 Stroke Unit, Hospital Universitario Ramon Y Cajal, Madrid, ESP

**Keywords:** frank’s sign, cerebrovascular diseases, brain swelling, cardiovascular diseases, diagonal earlobe crease

## Abstract

This case highlights the case of a woman in her 50´s and the association of Frank’s sign with vascular disease. Earlobe crease or Frank's sign is a diagonal crease in the earlobe that extends diagonally from the tragus to the edge of the auricle with an angle of approximately 45°. Its presence increases with advancing age and is thought to be predictive of vascular disease. The recognition of this sign is considered a key factor in the identification of patients with high vascular risk and endothelial dysfunction. The association with a reperfusion syndrome, like our patient, is not known.

## Introduction

Earlobe creases are surrogate markers for a high risk of cardiovascular disease [1]. There are no data concerning earlobe creases among stroke patients treated with reperfusion therapies. Malignant middle cerebral artery infarction is a devastating condition with high mortality and tissue damage that can happen in stroke patients. Vascular endothelial impairment might be involved in brain swelling in patients with an ischemic stroke, just like in earlobe creases. This case is illustrative because the two conditions are well-documented.

## Case presentation

**Figure 1:**
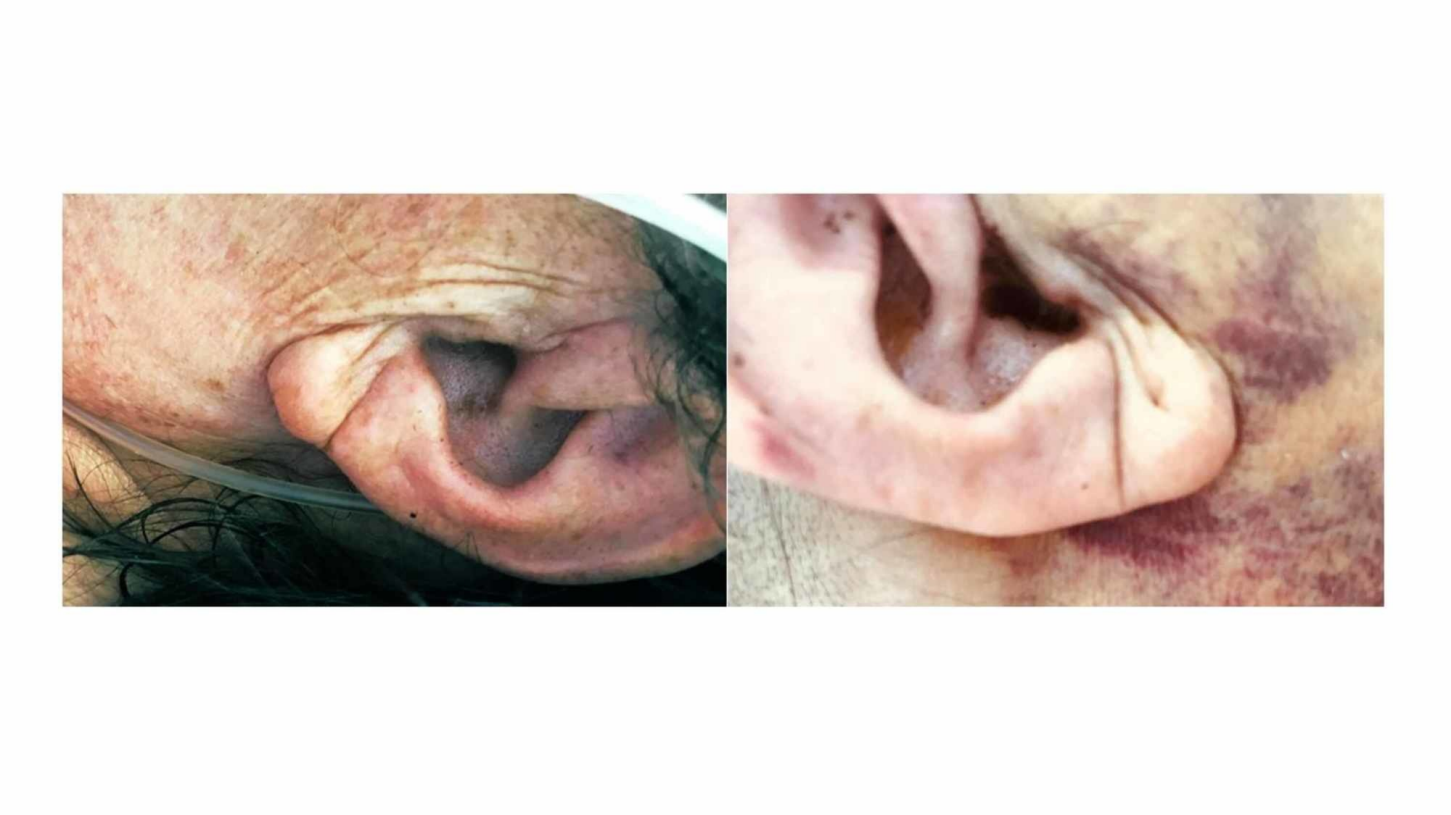
Photographs of the patient showing bilateral earlobe crease Earlobe crease appearing as a wrinkle extending from the tragus to the outer border of the earlobe on both sides

**Figure 2:**
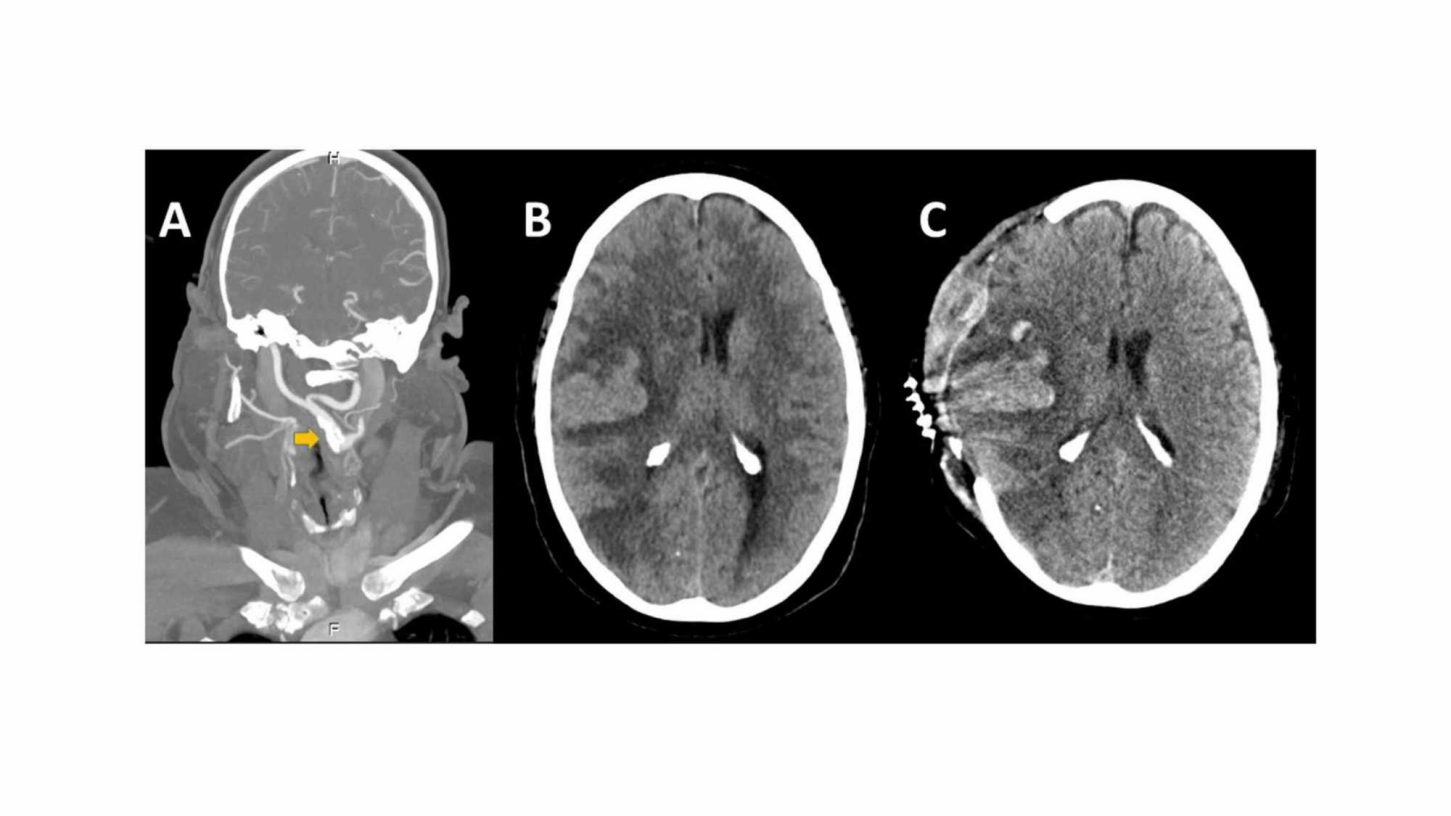
Computed tomography angiogram (CTA) of the extracranial and intracranial arteries (panel A), the non-contrast CT scan showing brain swelling (B), and after decompressive surgery (C) Atherosclerosis is seen on the right carotid segments (yellow arrow) and an acute ischemic lesion affecting the right middle cerebral artery (MCA) territory infarct with extensive brain edema and midline shift before (B) and after decompressive surgery (C).

A woman in her 50’s with a past medical history of hypertension, diabetes, and hypercholesterolemia presented to the emergency department with a five-hour history of left hemiplegia. Bilateral earlobe creases (Frank's sign) were noted in the Emergency Department and urgent multimodal imaging was performed with a computed tomography angiogram (CTA). Imaging revealed diffuse atherosclerosis in the extra- and intracranial arteries and a right middle cerebral artery occlusion. The patient was urgently transferred to the angiography suite for mechanical thrombectomy (M1 segment) (Figure 1). A mechanical thrombectomy was then performed with a stent retriever, the clot was completely removed, and the blood flow in the medium cerebral artery was completely restored. Within 24 hours of her treatment, she developed brain swelling, massive edema on the core of the infarction area, and urgent decompressive surgery was performed (Figure 2). The patient continued to experience severe left hemiparesis and disturbance of consciousness on Day 9 after surgery. She was transferred to another hospital for rehabilitation with persistent hemiplegia and a severe degree of disability (Modified Rankin Scale score 5 points).

Bilateral earlobe crease (ELC) or Frank’s sign could be seen on the patient [1]. Frank's sign is described as a diagonal crease in the earlobe that extends diagonally over the edge of the auricle with an angle of approximately 45°. It can be easily noticed at clinical examination and its presence increases with age and diabetes. Several studies have shown an association between ELC, cardiovascular disease, and non-lacunar ischemic stroke, supporting the notion that ELC could be a sign of longstanding atherosclerosis [2]. The presence of microvascular damage in histological studies on ELC and its relationship with carotid intima-media thickness (IMT) may support the hypothesis that ELC is associated with endothelial dysfunction and atherosclerosis [3]. 

## Discussion

Malignant middle cerebral artery infarction is a devastating condition with high mortality and tissue damage. Edema occurs in a large portion of the ischemic territory that leads to dysfunction of the tight junctions and the breakdown of the blood-brain barrier (BBB) [4]. Vasogenic brain edema occurs, resulting in space-occupying brain swelling. Patients with atherosclerosis and diffuse leukopathy have a decreased and declining capacity to regulate blood flow that can contribute to a malignant infarction. The exact mechanism through which ischemic injury disrupts the BBB is not fully understood, but active pinocytosis by endothelial damage appears to occur in this scenario. This may contribute to the flow of water through the ruptured BBB [4]. The case is illustrative because an external sign of vascular damage, like ELB, might be a marker of potential brain swelling in patients with ischemic stroke treated with reperfusion techniques. Patients with ischemic stroke and Frank's sign should be monitored closely, undergo a diagnostic workup to detect vascular disease, and implement preventative therapies. The sign can identify those who are at higher risk of brain swelling; however, the hypothesis needs to be confirmed with larger samples

## Conclusions

The association with ischemic stroke has been reported previously and the recognition of this signal is considered a critical factor in the identification of patients with high vascular risk. As with our patient, it could also be considered a sign for endothelium damage in patients treated with reperfusion therapies; however, larger samples are needed.
